# Transgender health objectives of training for adult Endocrinology and Metabolism programs: Outcomes of a modified-Delphi study

**DOI:** 10.1371/journal.pone.0301603

**Published:** 2024-05-20

**Authors:** Tehmina Ahmad, Leighton Schreyer, Raymond Fung, Catherine Yu

**Affiliations:** 1 Division of Endocrinology and Metabolism, Department of Medicine, University of Toronto, Toronto, ON, Canada; 2 Toronto Western Hospital, University Health Network, Toronto, ON, Canada; 3 Temerty Faculty of Medicine, University of Toronto, Toronto, ON, Canada; 4 Michael Garron Hospital, Toronto East Health Network, East York, ON, Canada; 5 St. Michael’s Hospital, Unity Health, Toronto, ON, Canada; Queens University - Canada, CANADA

## Abstract

**Background:**

Transgender people encounter significant barriers when seeking timely, high-quality healthcare, resulting in unmet medical needs with increased rates of diabetes, asthma, chronic obstructive pulmonary disease, and HIV. The paucity of postgraduate medical education to invest in standardization of transgender health training sustains these barriers, leaving physicians feeling unprepared and averse to provide transgender health care. Closing this education gap and improving transgender healthcare necessitates the development of consensus-built transgender health objectives of training (THOOT), particularly in Adult Endocrinology and Metabolism Residency programs.

**Methods:**

We conducted a two-round modified-Delphi process involving a nationally representative panel of experts, including Adult Endocrinology and Metabolism program directors, physician content experts, residents, and transgender community members, to identify THOOT for inclusion in Canadian Endocrinology and Metabolism Residency programs. Participants used a 5-point Likert scale to assess THOOT importance for curricular inclusion, with opportunities for written feedback. Data was collected through Qualtrics and analyzed after each round.

**Findings:**

In the first Delphi round, panelists reviewed and rated 81 literature extracted THOOT, achieving consensus on all objectives. Following panelists’ feedback, 5 THOOT were added, 9 removed, 34 consolidated into 12 objectives, and 47 were rephrased or retained. In the second Delphi round, panelists assessed 55 THOOT. Consensus was established for 8 THOOT. Program directors’ post-Delphi feedback further consolidated objectives to arrive at 4 THOOT for curriculum inclusion.

**Conclusions:**

To our knowledge, this is the first time a consensus-based approach has been used to establish THOOT for any subspecialty postgraduate medicine program across Canada or the United States. Our results lay the foundation towards health equity and social justice in transgender health medical education, offering a blueprint for future innovations.

## Introduction

In healthcare settings, transgender patients often face harassment, discrimination, and limited access to quality care, resulting in higher health service use for mental health and self-harm, and greater experiences of chronic physical conditions such as diabetes, asthma, chronic obstructive pulmonary disease, and HIV [1–4]. Despite the globally growing number of transgender individuals (0.3–4.5% in adults, 2.5–8.4% in children and adolescents) with specific healthcare needs, medical education has largely overlooked and neglected to prioritize standardized transgender health training, further exacerbating barriers to healthcare access [1–3, 5–7]. A recent literature review of transgender health education across undergraduate and graduate, allopathic and osteopathic medical schools in North America revealed that transgender health has yet to gain widespread curricular exposure [8]. Where transgender health education exists, it largely constitutes one-time attitude and awareness-based interventions that may offer short-term benefits but lack methodological robustness and long-term benefits [8]. The lack of transgender health education is especially pertinent at the postgraduate level in Endocrinology and Metabolism subspecialty programs, given the significant role endocrinologists play in directing transgender health hormonal care [9, 10]. In a 2017 American survey of Endocrinology fellows, 41.1% reported that their program did not provide dedicated transgender care content, and of those who reported training, 40% had received less than 2 hours of training content per year [9]. The inadequacy of postgraduate training has a ripple effect that ultimately results in clinicians being unable, unwilling, or uncomfortable with providing transgender care [11].

Importantly, educational interventions in transgender care enhance the ability of trainees to meaningfully address transgender patients’ healthcare needs. Multiple studies have shown an improvement in trainees’ knowledge, attitudes, and willingness to address transgender healthcare needs when they are exposed to didactic learning and standardized clinical encounters with transgender patients throughout their education [9, 12–14].

Closing the transgender health education gap necessitates the development of consensus-built Transgender Health Objectives of Training (THOOT) in Endocrinology and Metabolism Residency programs. Using Kern’s six-step model of curricular design (S1 Fig) [15], we previously conducted a scoping review to 1) identify the problem, and 2) target a needs assessment to better understand the current state of transgender health medical education [16]. While there are a breadth of studies on literature identified THOOT, there is currently no consensus on a validated, standardized set of THOOT [16]. To facilitate curriculum development and address steps 3 and 4 of Kern’s model (goals and objectives, and educational strategies), we surveyed an expert panel of Adult Endocrinology and Metabolism program directors, physician content experts, resident trainees, and transgender community members. We used a two-step modified Delphi process to achieve consensus on literature-extracted THOOT for curricular integration in Adult Endocrinology and Metabolism Residency programs across Canada. Our study lays a critical foundation towards health equity and social justice in postgraduate medical education, offering a blueprint for future curricular innovation.

## Methods

### Modified Delphi process

**Fig 1:**
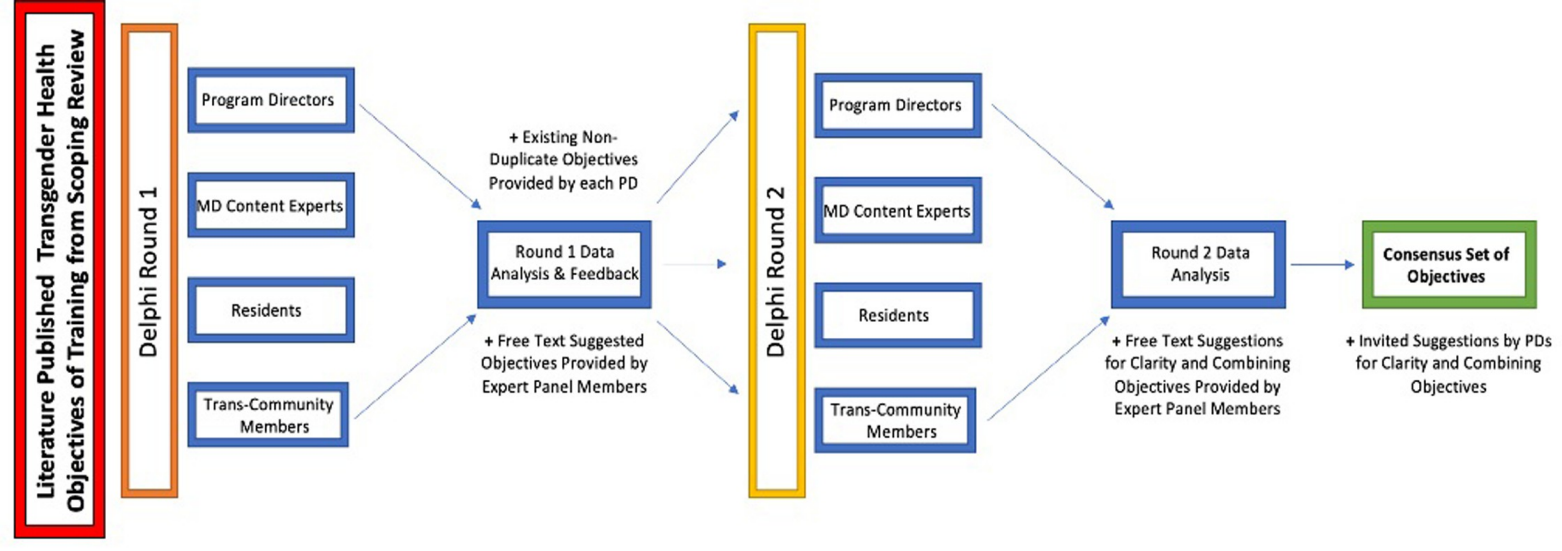
Overview of modified-Delphi process. Delphi rounds developing transgender health objectives of training.

We implemented a two-round modified Delphi technique [17–19] from January 2, 2023 to April 10, 2023 with weekly email reminders for each survey [20] to reach consensus on the inclusion of literature-identified THOOT [16] in Adult Endocrinology and Metabolism Residency programs across Canada. A modified Delphi method is a well-established strategy used to achieve consensus when there is limited evidence (S1 Table) [19, 21]. Following the Guidance on Conducting and REporting DElphi Studies (CREDES) (S2 Table) [19], two Delphi rounds were conducted (Fig 1) using questionnaires via a web-based survey system Qualtrics (Qualtrics XM, Version 12, Provo, Utah).

Ethical approval was obtained through the University of Toronto Research and Ethics Board protocol #34537.

### Delphi expert panelist recruitment

We identified four stakeholder groups as critical to include in the panel of experts: (a) Adult Endocrinology and Metabolism program directors, (b) physicians experienced in providing transgender health care (i.e., physician content experts), (c) Adult Endocrinology and Metabolism residents at any stage of training, and (d) transgender community members.

We invited each of the 14 program directors of Adult Endocrinology and Metabolism Residency programs in Canada by email to take part in the study, and further asked their administrative coordinators to forward a templated recruitment email and sign-up to physicians in their network who would be suitable to participate as physician content experts. Residents were recruited at the December 2022 National Academic Half-Day, delivered virtually through the University of British Columbia’s Division of Endocrinology. Research team members (TA & CY) used their professional networks to recruit transgender community members and further identify physician content experts for invitation to the study. No patients or personal relations of the research team were invited to participate as transgender community members to mitigate risk of bias and prevent power imbalances from influencing participants decision to participate in the study and/or their survey responses. Rather, transgender community members were invited to participate through collaborative alliances with 2SLGBTQIA+ health professional networks, and online community organizations.

To ensure national representation, we recruited 6 physician content experts and 6 residents, 2 each from western, central, and eastern Canada for both of these stakeholder groups, and 3 transgender community members, one each from western, central, and eastern Canada with diverse lived experience.

All participants received financial compensation for their time, in accordance with Canadian Institutes of Health Research (CIHR) and University of Toronto compensation and reimbursement of research participants standards. In addition, program directors, physician content experts, and residents received 3 credits per hour of participation to contribute to Section 3 of the Royal College of Physicians and Surgeons of Canada Maintenance of Certification (MOC) Program.

### Delphi round 1

Our first structured online questionnaire (Survey 1) consisted of the 81 THOOT that we identified in our previous scoping review [16], organized into 5 themes and sub-categorized as either ‘knowledge’, ‘skills’, or ‘attitude’ objectives [16]. Panelists provided written consent by agreeing to participate in the online survey. Panelists were asked to score the relative importance of each THOOT on a 5-point Likert scale (1 = not important, can be omitted; to 5 = essential) for curricular inclusion in a 2-year Endocrinology and Metabolism Residency program. For each theme, panelists were given the opportunity to provide free-text comments to suggest revisions to each THOOT and to suggest any additional THOOT felt suitable for inclusion. Program directors were asked to list any existing THOOT that were not presented but currently taught in their training programs.

Responses to Survey 1 were analyzed over a 4-week period. Two consensus criteria were set a priori: a) calculated mean score of 4 or higher, and b) scores of 4 or 5 from 70% of panelists, consistent with previous Delphi studies [18, 22–24].

### Delphi round 2

**Fig 2:**
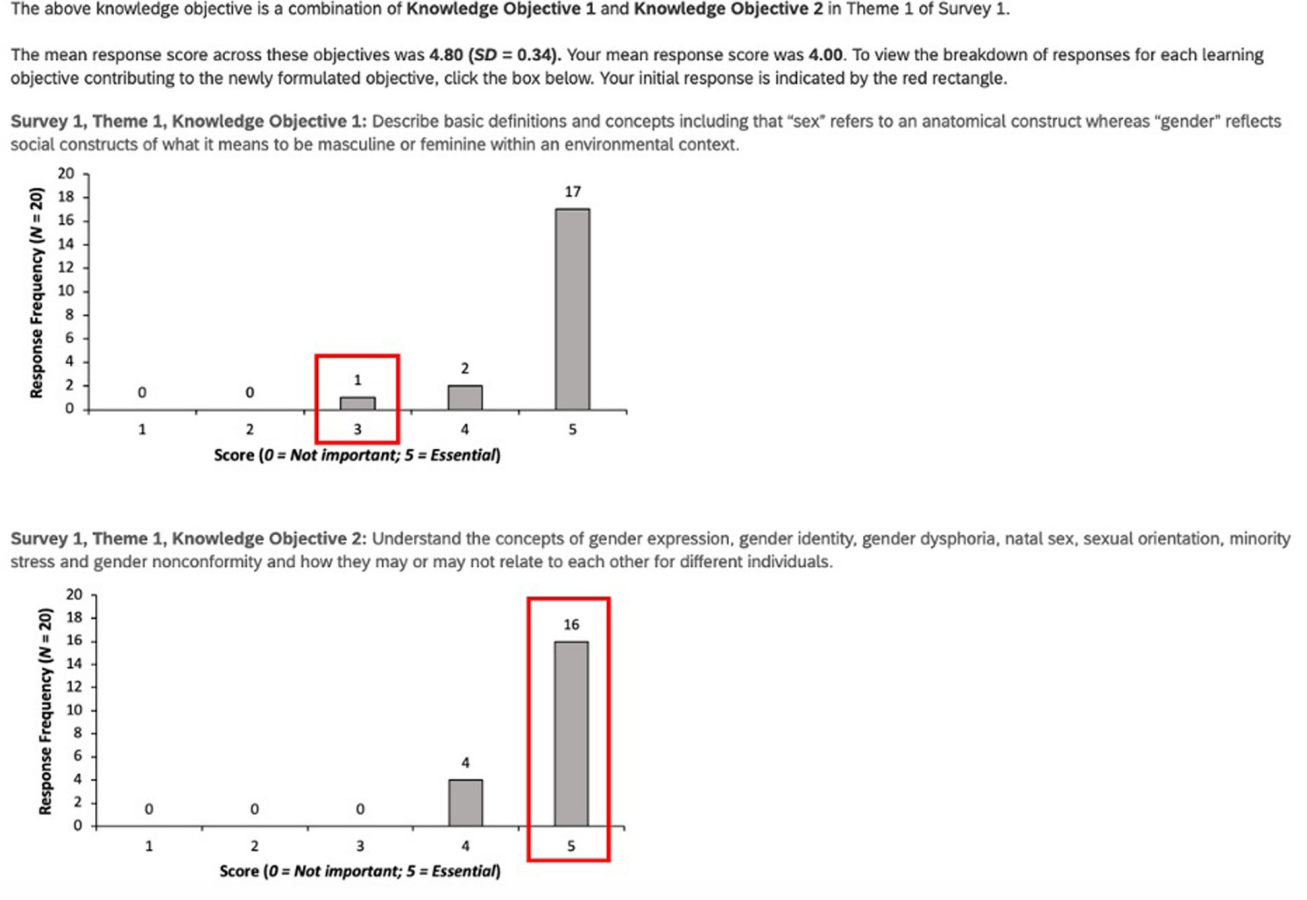
Example of graphically synthesized distribution score for each THOT. From Survey 1 the majority response is indicated by height of the shaded grey histogram, and the individual’s initial response indicated by the red box.

In the second round (Survey 2), each individual participant reviewed the new set of THOOT alongside graphically synthesized distribution scores for each THOOT from Survey 1 to help inform their decision-making (Fig 2). When THOOT were revised as combined, the panelists received their own and the groups’ aggregate mean scores and standard deviations with the option to review the individual scores for each original THOOT comprising the new THOOT. In addition to Likert scoring each THOOT, we also asked panelists to rank-order the THOOT within each of the 5 themes from most to least important for curricular inclusion to reduce anticipated courtesy bias and allow for further refinement of consensus.

Responses to Survey 2 were analyzed over a 4-week period. Two consensus criteria were set post-hoc to enhance the strength of the final list of objectives for curricular inclusion: a) scores of 4 or 5 from 100% of panelists, and b) achieving top-half rank from 70% of panelists [23–25]. We also conducted one-way analysis of variance (ANOVA) (Microsoft Excel 2019, Microsoft, Redmond, Washington) for each THOOT to assess for within- and between-group differences in scores.

At the end of Survey 2, we presented panelists with results from a study [26] suggesting that at least 35-hours of training are required to become competent in transgender care and asked panelists to select one of four categorical response options (’Less than 35 hours’, ‘35 hours’, ‘35–60 hours’, ‘More than 60 hours’), which they felt would be feasible to implement in a 2-year Endocrinology and Metabolism Residency program. We also asked panelists to review and rank-order a list of teaching methods (S2 Fig) from most to least effective for transgender health training. For both training hours and teaching methods, participants had the opportunity to provide free-text comments or suggestions to explain their response.

### Post-Delphi round 2 feedback

Following the second Delphi round, program directors were emailed a preliminary list of THOOT for which consensus was achieved and invited to provide final comments, suggestions, and revisions for clarity and readability of the consensus driven THOOT. Physician content experts, residents, and transgender community members were not included in this stage of the study since, at this time, feedback was specifically focused on advancing program uptake, rather than the content of the objectives themselves.

### Qualitative analysis

In the first Delphi round, each participant had 14 opportunities to provide feedback, with program directors given one additional survey opportunity to add any existing THOOT from their programs into the study. This allowed for 285 opportunities to comment or suggest changes to the objectives across the 20 completed surveys. In the second Delphi round, each participant had 10 opportunities to add comments or suggestions, for a total of 200 opportunities. Two research members (TA & LS) performed retrospective review of the second Delphi survey and independently analyzed responses using conventional content analysis [27, 28]. Selective coding occurred in the final stage of analysis, allowing for the development of overarching themes [27–29]. Disagreements were resolved through consensus discussion between team members (TA, LS, & CY). The results of the qualitative analysis are reported separately.

## Findings

### Expert panel

Of the 14 program directors invited and contacted, 5 agreed to participate; for both physician content experts and residents, we invited the first 2 people from each of western, central, and eastern Canada who expressed their interest in participating, all of whom agreed. Similarly, one transgender community member from western, central, and eastern Canada who expressed interest in participating was invited to the expert panel and all agreed. As such, our final panel comprised 20 experts (Table 1). All panelists completed both Delphi rounds (100% response rate).

**Table 1:** Demographic characteristics of panelists in the modified-Delphi study.

	Panelists (n = 20)
**Role**	
Endocrinology and Metabolism Program Director	5
Physician Content Expert	6
Endocrinology and Metabolism Resident	6
Transgender Community Member	3
**Geographic Region**	
Western Canada (British Columbia, Alberta, Saskatchewan)	6
Central Canada (Manitoba, Ontario, Quebec)	9
Eastern Canada (Maritime provinces, Newfoundland & Labrador)	5

### Consensus-building to select and refine THOOT for curricular inclusion

**Fig 3:**
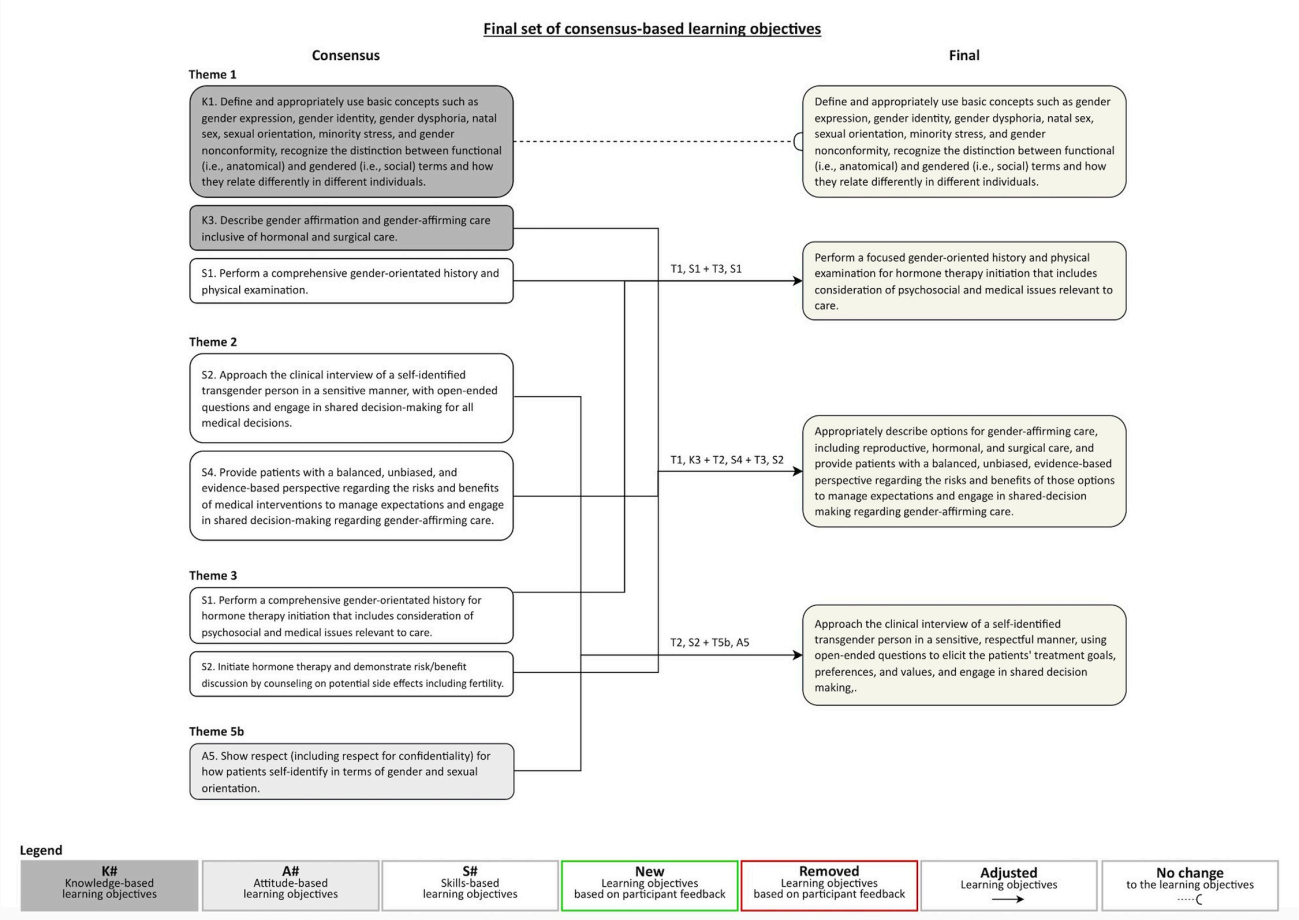
Final consensus-based list of transgender health objectives of training. For incorporation into Adult Canadian Endocrinology and Metabolism Residency programs.

At the end of round 1, the panel reached consensus, as defined a priori, on all 81 THOOT (100%). Based on panelists’ comments, which included suggestions around terminology and grammar to improve readability and consistency across objectives, as well as suggestions to aggregate or remove objectives that were perceived as overlapping, 5 objectives were added, 9 objectives were removed, and 34 objectives were combined into 12 objectives, resulting in a total of 55 THOOT to be re-entered into the second Delphi round (Fig 3).

At the end of round 2, the panel reached consensus on 8 THOOT, defined by the more stringent consensus criteria set following analysis of Survey 1. The post-Delphi feedback we received from program directors on these 8 consensus based THOOT led to further aggregation of objectives for clarity and concision, such that a final set of 4 THOOT were identified for integration into Canadian Endocrinology and Metabolism Residency programs (Fig 3). ANOVA analysis revealed no significant within- or between-group differences in the score of any THOOT (S1 File).

### Training hours and teaching method preferences

**Fig 4:**
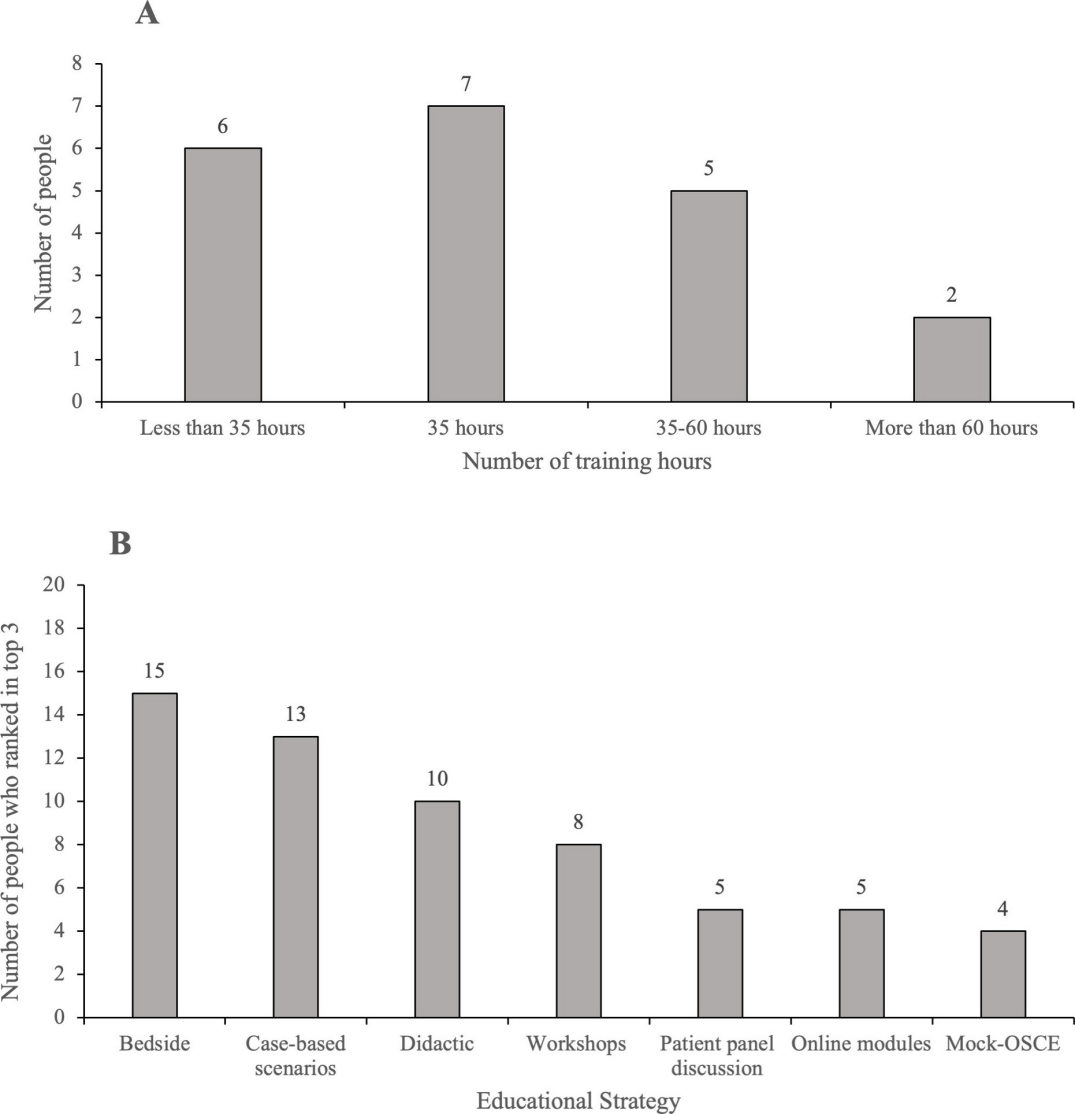
a. Panelists responses regarding number of hours transgender health content should be included in the curriculum. b. Panelists favoured educational strategy for which transgender health topics should be delivered.

The majority of panelists felt that, for a 2-year Endocrinology and Metabolism Residency program, at least 35 hours of training should be dedicated to transgender health training (Fig 4A). Of the 7 teaching methods presented, there was a strong preference for bedside teaching across all stakeholder groups, ranging from 66%-83% with a greater preference from learners ranking this within the top 3 teaching methods, (Fig 4B). Case-based scenarios and didactic lectures were also rated in the top 3 teaching methods, with preference for case-based scenarios ranging from 66–80% across stakeholder groups, and preference for didactic lectures ranging from 33–50% across stakeholder groups.

## Discussion

This study presents a consensus-building effort to identify Transgender Health Objectives of Training (THOOT) for integration into Adult Endocrinology and Metabolism Residency programs in Canada. The result is an evidence-driven list of 4 THOOT that, if implemented, may help reduce the transgender health education gap, thereby improving access to high-quality, appropriate healthcare for transgender people and reducing the disproportionate health disparities transgender people face [30, 31].

While previous studies have explored short-term development and application of a myriad of THOOT [16], this is the first study to use a modified-Delphi method to engage a diverse panel of experts to methodically arrive at a standardized set of nationally applicable THOOT for lasting curricular integration. This approach ensures that our results offer a comprehensive, validated set of THOOT that represent the collective expertise and perspectives of key stakeholders.

Under the framework of Kern’s six-step model of curricular design, [15] our study has addressed step 3 (goals and objectives) and suggested step 4 (educational strategies). Further work, namely step 5 (implementation), involves local uptake by program directors and integrating transgender health into didactic lectures across the curriculum. Nationally, our study provides a scaffold for the Royal College of Physicians and Surgeons Canada to build an evaluation process (step 6) as Endocrinology and Metabolism programs prepare for Competency Based Medical Education (CBME) in 2025.

As the THOOT we identified in this study are incorporated into Endocrinology and Metabolism Residency programs in Canada, it will be important to evaluate their effectiveness in enhancing trainees’ knowledge, attitudes, and skills in transgender healthcare. This evidence-based approach will help refine and further develop the curricula, ensuring their relevance, impact, and continuous improvement over time. As part of these evaluation efforts, it will be important to also assess the effectiveness of different training delivery methods. While our results offer insight into stakeholders’ preferences, there is, to our knowledge, currently no consensus on the most suitable teaching formats or the effectiveness of different educational interventions [32]. However, existing literature supports educational efforts to move towards longitudinally integrated, clinical skills based pedagogical interventions [8, 32]. Formal adoption of the proposed THOOT into Endocrinology and Metabolism Residency programs would mark a critical step towards health equity and social justice in medical education [33].

Building on the success of this study, future directions could involve establishing advanced areas of focused competency or specialized Transgender Medicine Fellowship programs. Notably, many training objectives were felt to be important but appropriately eliminated based on the stringent criteria used and relevance to core Endocrinology and Metabolism residency training; these additional training objectives could provide a foundation for forming an area of focused competence or Transgender Medicine Fellowship program in Canada. Future studies should also engage stakeholders from disciplines beyond endocrinology, such as primary care, psychiatry, emergency medicine, paediatrics, obstetrics and gynecology, surgery, and clinical fields of psychotherapy and speech language pathology, to further develop a more comprehensive and coordinated approach to transgender health.

Despite our best efforts to engage key stakeholders in this process and consider curricular limitations, we anticipate that there will still be barriers to implementation. That only 5 of 14 the program directors were willing to participate in this study, is suggestive of the prevailing de-prioritization of transgender health education [34]. Additional barriers to increasing transgender health exposure include limited curricular time, lack of topic-specific competency among faculty, endocrinologist availability, regional differences in acceptance of ideas, poor institutional support, and systemic bias as identified by Dubin et al. [8]. However, we believe that, with the anticipated transition to CBME in 2025 and the 2025 revision of the CanMEDS physician competency framework, which emphasizes themes of equity, diversity and inclusivity, and social justice [33, 35], there is further impetus to advocate for and, indeed, begin implementing THOOT in Adult Endocrinology and Metabolism Residency programs.

Beyond identifying a series of consensus-based, evidence-informed THOOT for integration into Adult Endocrinology and Metabolism Residency programs in Canada, our study provides a blueprint approach for consensus-modelling in medical education using an equity, diversity, and inclusion lens that can be easily replicated in different contexts to develop a locally informed curriculum.

### Limitations

While use of a modified-Delphi technique helped reduce the effect of panelists’ status on results, and adhered to the CREDES Guidance on Conducting and REporting DElphi Studies, [17, 19, 23, 36] panelists’ scoring of THOOT may still have been influenced by courtesy bias. Panelists may have felt compelled to provide more socially desirable responses, which may have impacted the objectivity of their ratings and may account for why, after the first Delphi round, all THOOT met the consensus criteria defined a priori. To help counter any effects of courtesy bias, we set a higher threshold for absolute consensus and, in addition to scoring THOOT, we also asked panelists to rank THOOT in the second Delphi round to ascertain relative importance.

The long list of THOOT included in the first Delphi round offers insight into the lack of a standardized approach to transgender health training to date, and the complexity of identifying a small, yet comprehensive list of objectives for curricular integration. Even so, our reliance on existing literature extracted THOOT may have introduced publication bias. However, given the extensive list of THOOT we began with, and considering that many of these objectives had overlapping content, we believe that any unpublished THOOT not identified in our literature search would be unlikely to have a significant effect on our results. While the length of the initial THOOT list may have introduced the risk of cognitive fatigue, the 100% response rate across both surveys demonstrates that panelists remained engaged throughout the process. Since our focus was on Canadian Endocrinology and Metabolism Residency programs, the generalizability of our findings to other regions may be limited, as THOOT may vary across different healthcare and cultural contexts.

## Conclusions

Our study addresses a critical gap in medical education by developing a standardized set of consensus based THOOT for incorporation into Adult Canadian Endocrinology and Metabolism Residency programs. Beyond helping to ensure that future endocrinology graduates are able to provide high-quality, affirming healthcare to transgender individuals, incorporating these THOOT will have a legacy effect, where providing transgender care will become part of the baseline competencies expected of hormone specialists.

## Supporting information

S1 Fig Kern’s six-step model of curricular design. (TIFF)

S2 Fig Panelists asked to rank-order teaching methods felt to be most and least effective for transgender health training. (TIFF)

S3 Fig Evolution of transgender health training objectives organized by theme using modified-Delphi consensus. (TIFF)

S1 Table Modified-Delphi survey method justification. (DOCX)

S2 Table Recommendations for the Conducting and Reporting of Delphi Studies (CREDES) checklist. (DOCX)

S1 File ANOVA analysis demonstrating no within- and between-group differences. (PDF)

S1 Raw data Raw data & distribution of responses. (XLSX)
