# Supplementary figures and images for: Transgender health objectives of training for adult Endocrinology and Metabolism programs: Outcomes of a modified-Delphi study
Source: PLoS One. 2024 May 20;19(5):e0301603. doi: 10.1371/journal.pone.0301603 (PMC11104599; doi:10.1371/journal.pone.0301603)

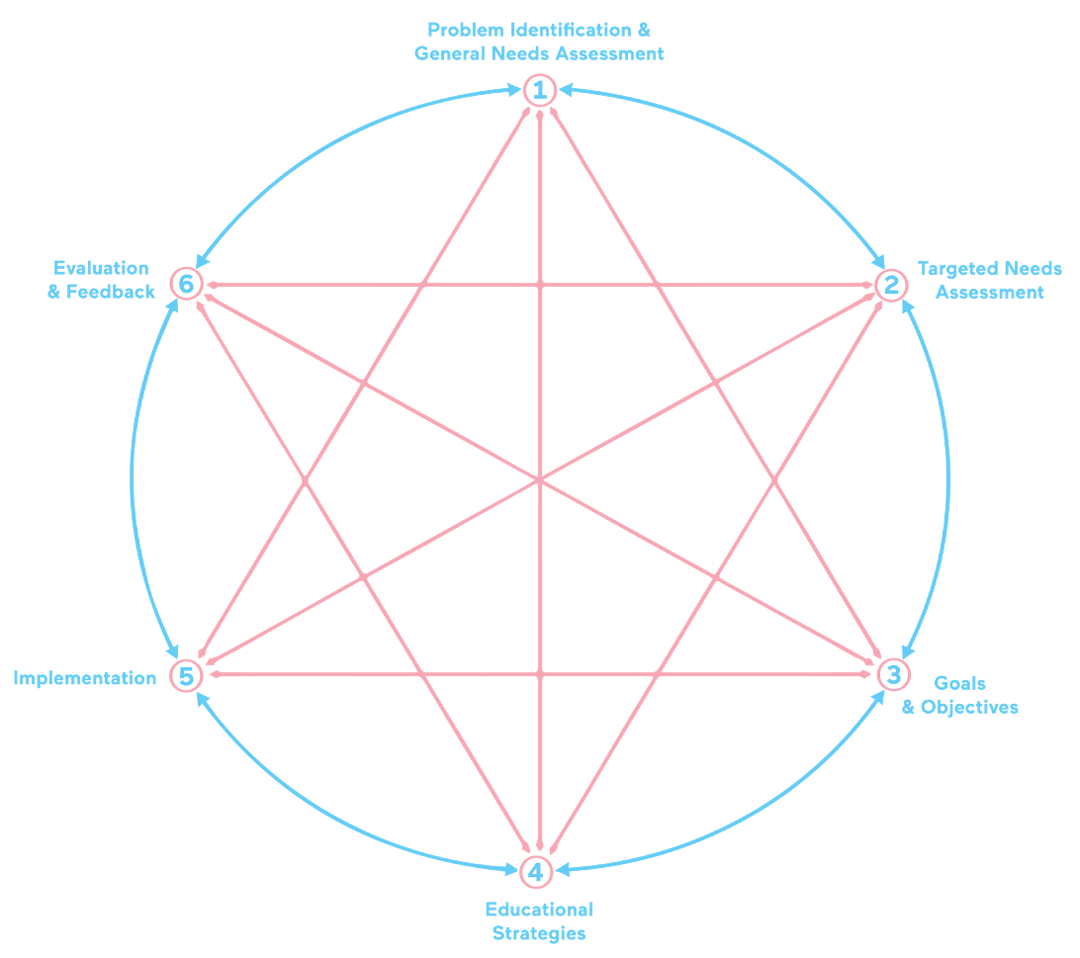

Supplement: S1 Fig — (TIFF) [file pone.0301603.s001.tiff]

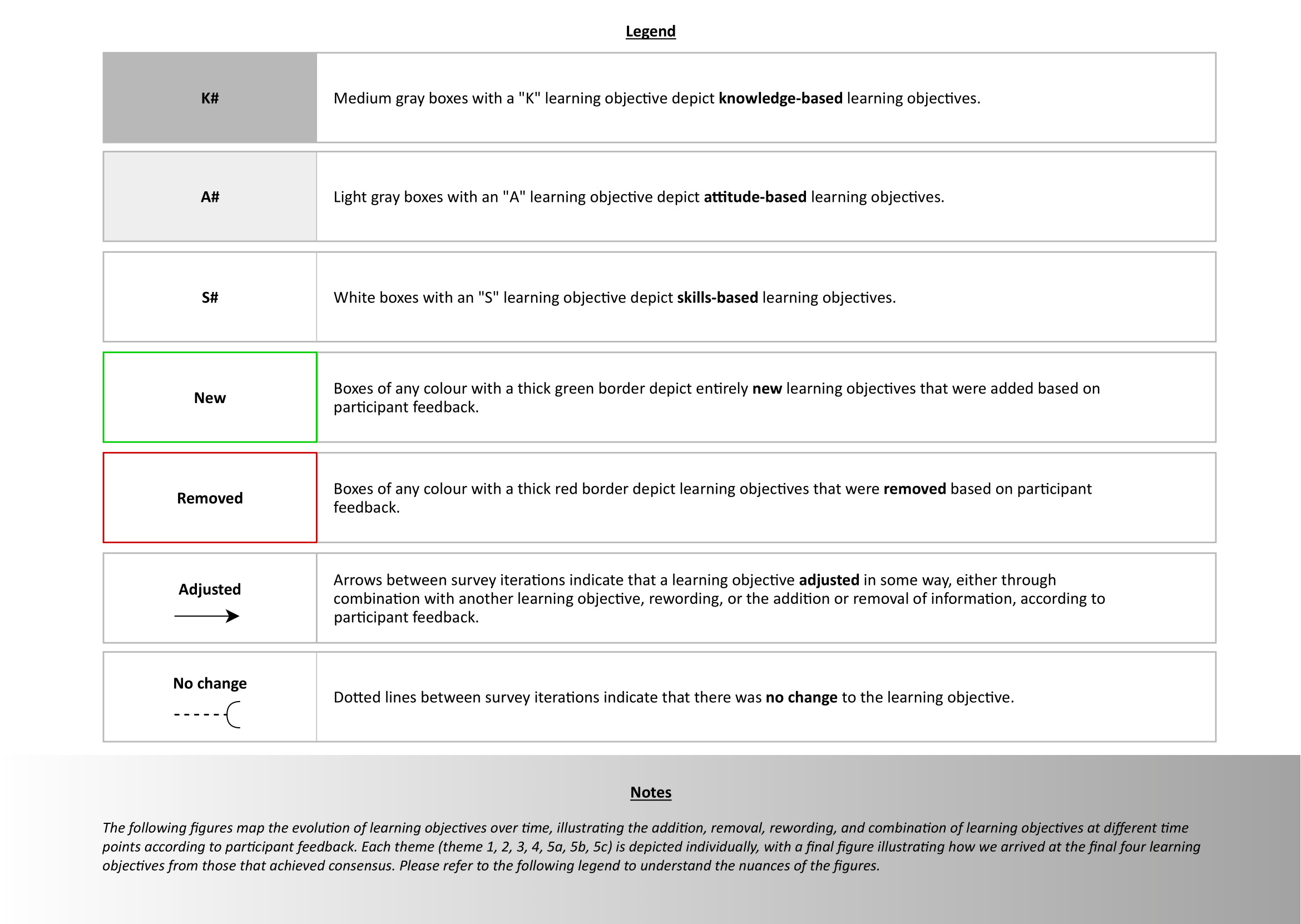

Supplement: S3 Fig — (TIFF) [file pone.0301603.s003.tiff]
