# Supplementary material for: Transgender health objectives of training for adult Endocrinology and Metabolism programs: Outcomes of a modified-Delphi study
Source: PLoS One. 2024 May 20;19(5):e0301603. doi: 10.1371/journal.pone.0301603 (PMC11104599; doi:10.1371/journal.pone.0301603)
Supplement: S1 Table — (DOCX) [file pone.0301603.s004.docx]

### **S1 Table. Modified-Delphi survey method justification.**

| Surveys are blinded for anonymity and each iterative cycle is conducted such that experts consider the results of the previous round and have the option of changing their answer if they choose.^1^ |
| --- |
| The ideal number of panellists for a Delphi is not set in stone, however, literature recommends between 10 to 20 participants to ensure arrival at consensus among experts.^2^{Veugelers, 2020 #41}{Veugelers, 2020 #41}{Veugelers, 2020 #41}{Veugelers, 2020 #41}{Veugelers, 2020 #41}{Veugelers, 2020 #41}{Veugelers, 2020 #41}{Veugelers, 2020 #41}{Veugelers, 2020 #41} |
| We selected each of these stakeholder groups for the following reasons: a) Program Directors have a direct impact in dictating content and shaping curricular design; b) Physician content experts draw from their direct clinical experiences with transgender patients, and translate this into education experiences for residents; c) Residents ensure there is a learner-centered approach to identifying knowledge gaps and areas that can be supported ; d) Transgender Community members are lived-experience experts who inform the clinical expertise of what should be known about their healthcare. |
| Involvement of experts from across the country who are working in various practice settings adds several unique perspectives, thus enhancing the likelihood that our findings will be pertinent and applicable to Endocrinology and Metabolism programs nationwide and to the transgender population we serve. |

**References**

1. Diamond IR, Grant RC, Feldman BM, et al. Defining consensus: a systematic review recommends methodologic criteria for reporting of Delphi studies. *Journal of clinical epidemiology.* 2014;67:401-9.
2. Veugelers R, Gaakeer MI, Patka P, Huijsman R. Improving design choices in Delphi studies in medicine: the case of an exemplary physician multi-round panel study with 100% response. *BMC Medical Research Methodology.* 2020;20:1-15.
