# Supplementary material for: Transgender health objectives of training for adult Endocrinology and Metabolism programs: Outcomes of a modified-Delphi study
Source: PLoS One. 2024 May 20;19(5):e0301603. doi: 10.1371/journal.pone.0301603 (PMC11104599; doi:10.1371/journal.pone.0301603)
Supplement: S1 File — (PDF) [file pone.0301603.s006.pdf]

LEE	*PCE*	*PD*	*R*
5	5	5	5
5	5	4	5
5	5	5	5
	5	5	4
	5	5	5
	5		4

Anova: Single Factor

SUMMARY

Groups	*Count*	*Sum*	*Average*	*Variance*
LEE	3	15	5	0
PCE	6	30	5	0
PD	5	24	4.8	0.2
R	6	28	4.66666667	0.26666667

ANOVA

ırce of Variati	*SS*	*df*	*MS*	*F*	*P-value*	*F crit*
Between Gro	0.41666667	3	0.13888889	1.04166667	0.40094496	3.23887152
Within Group	2.13333333	16	0.13333333			
Total	2.55	19				

LEE	*PCE*	*PD*	*R*
5	5	2	4
2	5	3	3
5	3	2	5
	4	5	5
	3	3	3
	4		4

Anova: Single Factor

SUMMARY

Groups	*Count*	*Sum*	*Average*	*Variance*
LEE	3	12	4	3
PCE	6	24	4	0.8
PD	5	15	3	1.5
R	6	24	4	0.8

ANOVA

ırce of Variati	*SS*	*df*	*MS*	*F*	*P-value*	*F crit*
Between Gro	3.75	3	1.25	1	0.41823758	3.23887152
Within Groug	20	16	1.25			
Total	23.75	19				

LEE	PCE	PD	R
5	5	5	5
5	5	5	5
5	5	5	5
	5	5	4
	5	5	5
	5		5

Anova: Single Factor

SUMMARY

Groups	Count	Sum	Average	Variance
LEE	3	15	5	0
PCE	6	30	5	0
PD	5	25	5	0
R	6	29	4.83333333	0.16666667

ANOVA

urce of Variati	SS	df	MS	F	P-value	F crit
Between Gro	0.11666667	3	0.03888889	0.74666667	0.53994993	3.23887152
Within Group	0.83333333	16	0.05208333			
Total	0.95	19				

LEE	PCE	PD	R
5	5	2	4
2	5	5	4
5	4	4	3
	5	5	4
	1	5	4
	4		5

Anova: Single Factor

SUMMARY

Groups	Count	Sum	Average	Variance
LEE	3	12	4	3
PCE	6	24	4	2.4
PD	5	21	4.2	1.7
R	6	24	4	0.4

ANOVA

urce of Variati	SS	df	MS	F	P-value	F crit
Between Gro	0.15	3	0.05	0.02985075	0.99277737	3.23887152
Within Group	26.8	16	1.675			
Total	26.95	19				

LEE	*PCE*	*PD*	*R*
4	5	4	4
1	5	4	4
5	3	2	3
	2	5	4
	3	4	4
	4		5

Anova: Single Factor

SUMMARY

Groups	*Count*	*Sum*	*Average*	*Variance*
LEE	3	10	3.33333333	4.33333333
PCE	6	22	3.66666667	1.46666667
PD	5	19	3.8	1.2
R	6	24	4	0.4

ANOVA

ırce of Variati	*SS*	*df*	*MS*	*F*	*P-value*	*F crit*
Between Gro	0.95	3	0.31666667	0.22222222	0.87954054	3.23887152
Within Group	22.8	16	1.425			
Total	23.75	19				

LEE	*PCE*	*PD*	*R*
5	5	4	4
5	5	4	4
5	4	2	3
	4	5	5
	4	5	4
	4		5

Anova: Single Factor

SUMMARY

Groups	*Count*	*Sum*	*Average*	*Variance*
LEE	3	15	5	0
PCE	6	26	4.33333333	0.26666667
PD	5	20	4	1.5
R	6	25	4.16666667	0.56666667

ANOVA

ırce of Variati	*SS*	*df*	*MS*	*F*	*P-value*	*F crit*
Between Gro	2.03333333	3	0.67777778	1.06666667	0.39091296	3.23887152
Within Grour	10.1666667	16	0.63541667			
Total	12.2	19				

LEE	*PCE*	*PD*	*R*
5	5	5	5
5	5	5	5
5	5	5	5
	5	5	4
	5	5	5
	5		5

Anova: Single Factor

SUMMARY

Groups	*Count*	*Sum*	*Average*	*Variance*
LEE	3	15	5	0
PCE	6	30	5	0
PD	5	25	5	0
R	6	29	4.83333333	0.16666667

ANOVA

ırce of Variati	*SS*	*df*	*MS*	*F*	*P-value*	*F crit*
Between Gro	0.11666667	3	0.03888889	0.74666667	0.53994993	3.23887152
Within Group	0.83333333	16	0.05208333			
Total	0.95	19				

LEE	*PCE*	*PD*	*R*
5	5	4	4
4	4	4	5
5	4	4	4
	5	5	4
	2	4	4
	4		5

Anova: Single Factor

SUMMARY

Groups	*Count*	*Sum*	*Average*	*Variance*
LEE	3	14	4.66666667	0.33333333
PCE	6	24	4	1.2
PD	5	21	4.2	0.2
R	6	26	4.33333333	0.26666667

ANOVA

ırce of Variati	*SS*	*df*	*MS*	*F*	*P-value*	*F crit*
Between Gro	0.95	3	0.31666667	0.57575758	0.63920923	3.23887152
Within Grouբ	8.8	16	0.55			
Total	9.75	19				

LEE	*PCE*	*PD*	*R*
5	5	4	4
5	5	4	5
5	4	4	5
	4	4	4
	2	5	4
	4		5

Anova: Single Factor

SUMMARY

Groups	*Count*	*Sum*	*Average*	*Variance*
LEE	3	15	5	0
PCE	6	24	4	1.2
PD	5	21	4.2	0.2
R	6	27	4.5	0.3

ANOVA

ırce of Variati	*SS*	*df*	*MS*	*F*	*P-value*	*F crit*
Between Gro	2.25	3	0.75	1.44578313	0.26664546	3.23887152
Within Group	8.3	16	0.51875			
Total	10.55	19				

LEE	*PCE*	*PD*	*R*
5	5	5	5
2	5	5	5
5	4	5	5
	2	5	5
	5	5	5
	5		5

Anova: Single Factor

SUMMARY

Groups	*Count*	*Sum*	*Average*	*Variance*
LEE	3	12	4	3
PCE	6	26	4.33333333	1.46666667
PD	5	25	5	0
R	6	30	5	0

ANOVA

ırce of Variati	*SS*	*df*	*MS*	*F*	*P-value*	*F crit*
Between Gro	3.21666667	3	1.07222222	1.28666667	0.31289909	3.23887152
Within Grour	13.3333333	16	0.83333333			
Total	16.55	19				

LEE	*PCE*	*PD*	*R*
5	5	5	5
5	5	5	5
5	4	4	4
	3	4	4
	4	5	4
	4		5

Anova: Single Factor

SUMMARY

Groups	*Count*	*Sum*	*Average*	*Variance*
LEE	3	15	5	0
PCE	6	25	4.16666667	0.56666667
PD	5	23	4.6	0.3
R	6	27	4.5	0.3

ANOVA

urce of Variati	*SS*	*df*	*MS*	*F*	*P-value*	*F crit*
Between Gro	1.46666667	3	0.48888889	1.41365462	0.2753713	3.23887152
Within Grou	5.53333333	16	0.34583333			
Total	7	19				

LEE	*PCE*	*PD*	*R*
5	5	3	5
5	5	4	5
5	4	5	5
	4	5	4
	4	5	4
	4		5

Anova: Single Factor

SUMMARY

Groups	*Count*	*Sum*	*Average*	*Variance*
LEE	3	15	5	0
PCE	6	26	4.33333333	0.26666667
PD	5	22	4.4	0.8
R	6	28	4.66666667	0.26666667

ANOVA

ırce of Variati	*SS*	*df*	*MS*	*F*	*P-value*	*F crit*
Between Gro	1.08333333	3	0.36111111	0.98484848	0.42470701	3.23887152
Within Group	5.86666667	16	0.36666667			
Total	6.95	19				

LEE	PCE	PD	R
5	5	5	5
5	5	5	5
5	4	5	5
	4	5	5
	5	5	5
	5		5

Anova: Single Factor

SUMMARY

Groups	*Count*	*Sum*	*Average*	*Variance*
LEE	3	15	5	0
PCE	6	28	4.66666667	0.26666667
PD	5	25	5	0
R	6	30	5	0

ANOVA

ırce of Variati	*SS*	*df*	*MS*	*F*	*P-value*	*F crit*
Between Gro	0.46666667	3	0.15555556	1.86666667	0.17592912	3.23887152
Within Group	1.33333333	16	0.08333333			
Total	1.8	19				

LEE	*PCE*	*PD*	*R*
5	5	5	4
1	5	5	4
5	4	5	5
	2	4	5
	4	4	4
	5		5

Anova: Single Factor

SUMMARY

Groups	*Count*	*Sum*	*Average*	*Variance*
LEE	3	11	3.66666667	5.33333333
PCE	6	25	4.16666667	1.36666667
PD	5	23	4.6	0.3
R	6	27	4.5	0.3

ANOVA

ırce of Variati	*SS*	*df*	*MS*	*F*	*P-value*	*F crit*
Between Gro	2	3	0.66666667	0.52805281	0.66936199	3.23887152
Within Group	20.2	16	1.2625			
Total	22.2	19				

LEE	*PCE*	*PD*	*R*
5	5	5	5
5	5	5	5
5	5	5	5
	5	5	5
	5	5	5
	5		5

Anova: Single Factor

SUMMARY

Groups	*Count*	*Sum*	*Average*	*Variance*
LEE	3	15	5	0
PCE	6	30	5	0
PD	5	25	5	0
R	6	30	5	0

ANOVA

ırce of Variati	*SS*	*df*	*MS*	*F*	*P-value*	*F crit*
Between Gro	0	3	0	65535	#DIV/0!	3.23887152
Within Group	0	16	0			
Total	0	19				

LEE	*PCE*	*PD*	*R*
5	5	5	5
5	5	5	5
5	3	5	4
	5	5	4
	5	5	5
	5		5

Anova: Single Factor

SUMMARY

Groups	*Count*	*Sum*	*Average*	*Variance*
LEE	3	15	5	0
PCE	6	28	4.66666667	0.66666667
PD	5	25	5	0
R	6	28	4.66666667	0.26666667

ANOVA

urce of Variati	*SS*	*df*	*MS*	*F*	*P-value*	*F crit*
Between Gro	0.53333333	3	0.17777778	0.60952381	0.61849465	3.23887152
Within Group	4.66666667	16	0.29166667			
Total	5.2	19				

LEE	*PCE*	*PD*	*R*
5	5	5	5
5	5	5	5
5	4	4	5
	4	5	4
	5	5	4
	4		5

Anova: Single Factor

SUMMARY

Groups	*Count*	*Sum*	*Average*	*Variance*
LEE	3	15	5	0
PCE	6	27	4.5	0.3
PD	5	24	4.8	0.2
R	6	28	4.66666667	0.26666667

ANOVA

ırce of Variati	*SS*	*df*	*MS*	*F*	*P-value*	*F crit*
Between Gro	0.56666667	3	0.18888889	0.83180428	0.49573993	3.23887152
Within Grouբ	3.63333333	16	0.22708333			
Total	4.2	19				

LEE	PCE	PD	R
5	5	5	5
5	5	5	5
5	5	5	5
	5	5	3
	5	5	5
	5		5

Anova: Single Factor

SUMMARY

Groups	Count	Sum	Average	Variance
LEE	3	15	5	0
PCE	6	30	5	0
PD	5	25	5	0
R	6	28	4.66666667	0.66666667

ANOVA

ırce of Variati	SS	df	MS	F	P-value	F crit
Between Gro	0.46666667	3	0.15555556	0.74666667	0.53994993	3.23887152
Within Grour	3.33333333	16	0.20833333			
Total	3.8	19				

LEE	PCE	PD	R
5	5	5	5
5	5	5	5
5	5	5	5
	5	5	4
	5	5	5
	5		5

Anova: Single Factor

SUMMARY

Groups	Count	Sum	Average	Variance
LEE	3	15	5	0
PCE	6	30	5	0
PD	5	25	5	0
R	6	29	4.83333333	0.16666667

ANOVA

urce of Variati	SS	df	MS	F	P-value	F crit
Between Gro	0.11666667	3	0.03888889	0.74666667	0.53994993	3.23887152
Within Grouр	0.83333333	16	0.05208333			
Total	0.95	19				

LEE	*PCE*	*PD*	*R*
4	5	5	4
1	5	5	3
5	4	5	3
	3	5	3
	3	4	5
	5		5

Anova: Single Factor

SUMMARY

Groups	*Count*	*Sum*	*Average*	*Variance*
LEE	3	10	3.33333333	4.33333333
PCE	6	25	4.16666667	0.96666667
PD	5	24	4.8	0.2
R	6	23	3.83333333	0.96666667

ANOVA

ırce of Variati	*SS*	*df*	*MS*	*F*	*P-value*	*F crit*
Between Gro	4.66666667	3	1.55555556	1.30081301	0.30846666	3.23887152
Within Group	19.1333333	16	1.19583333			
Total	23.8	19				

LEE	*PCE*	*PD*	*R*
5	5	5	5
5	5	5	5
5	5	4	5
	5	5	4
	5	5	5
	5		5

Anova: Single Factor

SUMMARY

Groups	*Count*	*Sum*	*Average*	*Variance*
LEE	3	15	5	0
PCE	6	30	5	0
PD	5	24	4.8	0.2
R	6	29	4.83333333	0.16666667

ANOVA

ırce of Variati	*SS*	*df*	*MS*	*F*	*P-value*	*F crit*
Between Gro	0.16666667	3	0.05555556	0.54421769	0.65903095	3.23887152
Within Group	1.63333333	16	0.10208333			
Total	1.8	19				

LEE	*PCE*	*PD*	*R*
5	5	3	5
2	5	5	5
5	5	5	5
	5	5	4
	5	5	5
	5		5

Anova: Single Factor

SUMMARY

Groups	*Count*	*Sum*	*Average*	*Variance*
LEE	3	12	4	3
PCE	6	30	5	0
PD	5	23	4.6	0.8
R	6	29	4.83333333	0.16666667

ANOVA

urce of Variati	*SS*	*df*	*MS*	*F*	*P-value*	*F crit*
Between Gro	2.16666667	3	0.72222222	1.1517165	0.35863657	3.23887152
Within Group	10.0333333	16	0.62708333			
Total	12.2	19				

LEE	*PCE*	*PD*	*R*
5	5	5	5
5	5	5	5
5	5	5	4
	5	5	4
	5	5	5
	5		5

Anova: Single Factor

SUMMARY

Groups	*Count*	*Sum*	*Average*	*Variance*
LEE	3	15	5	0
PCE	6	30	5	0
PD	5	25	5	0
R	6	28	4.66666667	0.26666667

ANOVA

ırce of Variati	*SS*	*df*	*MS*	*F*	*P-value*	*F crit*
Between Gro	0.46666667	3	0.15555556	1.86666667	0.17592912	3.23887152
Within Grour	1.33333333	16	0.08333333			
Total	1.8	19				

LEE	PCE	PD	R
5	5	5	5
5	5	5	5
5	5	5	5
	5	5	4
	5	5	5
	5		5

Anova: Single Factor

SUMMARY

Groups	Count	Sum	Average	Variance
LEE	3	15	5	0
PCE	6	30	5	0
PD	5	25	5	0
R	6	29	4.83333333	0.16666667

ANOVA

ırce of Variati	SS	df	MS	F	P-value	F crit
Between Gro	0.11666667	3	0.03888889	0.74666667	0.53994993	3.23887152
Within Grouр	0.83333333	16	0.05208333			
Total	0.95	19				

LEE	*PCE*	*PD*	*R*
5	5	5	5
5	5	5	5
5	5	5	5
	5	5	5
	5	5	5
	5		5

Anova: Single Factor

SUMMARY

Groups	*Count*	*Sum*	*Average*	*Variance*
LEE	3	15	5	0
PCE	6	30	5	0
PD	5	25	5	0
R	6	30	5	0

ANOVA

ırce of Variati	*SS*	*df*	*MS*	*F*	*P-value*	*F crit*
Between Gro	0	3	0	65535	#DIV/0!	3.23887152
Within Grouŗ	0	16	0			
Total	0	19				

LEE	*PCE*	*PD*	*R*
5	5	2	5
1	4	5	5
5	4	5	4
	5	5	4
	4	4	4
	4		5

Anova: Single Factor

SUMMARY

Groups	*Count*	*Sum*	*Average*	*Variance*
LEE	3	11	3.66666667	5.33333333
PCE	6	26	4.33333333	0.26666667
PD	5	21	4.2	1.7
R	6	27	4.5	0.3

ANOVA

ırce of Variati	*SS*	*df*	*MS*	*F*	*P-value*	*F crit*
Between Gro	1.45	3	0.48333333	0.38095238	0.7680749	3.23887152
Within Grouɽ	20.3	16	1.26875			
Total	21.75	19				

LEE	*PCE*	*PD*	*R*
5	5	4	5
5	5	5	3
5	5	2	3
	4	5	3
	5	5	3
	5		5

Anova: Single Factor

SUMMARY

Groups	*Count*	*Sum*	*Average*	*Variance*
LEE	3	15	5	0
PCE	6	29	4.83333333	0.16666667
PD	5	21	4.2	1.7
R	6	22	3.66666667	1.06666667

ANOVA

ırce of Variati	*SS*	*df*	*MS*	*F*	*P-value*	*F crit*
Between Gro	5.58333333	3	1.86111111	2.29648672	0.11664872	3.23887152
Within Group	12.9666667	16	0.81041667			
Total	18.55	19				

LEE	PCE	PD	R
5	5	2	5
5	5	4	5
5	4	4	4
	2	5	4
	3	5	3
	4		5

Anova: Single Factor

SUMMARY

Groups	Count	Sum	Average	Variance
LEE	3	15	5	0
PCE	6	23	3.83333333	1.36666667
PD	5	20	4	1.5
R	6	26	4.33333333	0.66666667

ANOVA

ırce of Variati	SS	df	MS	F	P-value	F crit
Between Gro	3.03333333	3	1.01111111	1.00068729	0.41794644	3.23887152
Within Group	16.1666667	16	1.01041667			
Total	19.2	19				

LEE	*PCE*	*PD*	*R*
5	5	2	5
5	5	5	5
5	5	1	4
	2	5	4
	4	5	4
	5		5

Anova: Single Factor

SUMMARY

Groups	*Count*	*Sum*	*Average*	*Variance*
LEE	3	15	5	0
PCE	6	26	4.33333333	1.46666667
PD	5	18	3.6	3.8
R	6	27	4.5	0.3

ANOVA

urce of Variati	*SS*	*df*	*MS*	*F*	*P-value*	*F crit*
Between Gro	4.16666667	3	1.38888889	0.9246417	0.45139646	3.23887152
Within Group	24.0333333	16	1.50208333			
Total	28.2	19				

LEE	*PCE*	*PD*	*R*
5	5	1	4
5	5	5	4
5	3	4	4
	1	5	4
	1	4	3
	3		5

Anova: Single Factor

SUMMARY

Groups	*Count*	*Sum*	*Average*	*Variance*
LEE	3	15	5	0
PCE	6	18	3	3.2
PD	5	19	3.8	2.7
R	6	24	4	0.4

ANOVA

ırce of Variati	*SS*	*df*	*MS*	*F*	*P-value*	*F crit*
Between Gro	8.4	3	2.8	1.55555556	0.23897039	3.23887152
Within Group	28.8	16	1.8			
Total	37.2	19				

LEE	PCE	PD	R
5	5	1	5
3	5	5	5
5	3	4	5
	1	5	4
	1	5	3
	3		5

Anova: Single Factor

SUMMARY

Groups	Count	Sum	Average	Variance
LEE	3	13	4.33333333	1.33333333
PCE	6	18	3	3.2
PD	5	20	4	3
R	6	27	4.5	0.7

ANOVA

ırce of Variati	SS	df	MS	F	P-value	F crit
Between Gro	7.63333333	3	2.54444444	1.19154472	0.34446514	3.23887152
Within Grouբ	34.1666667	16	2.13541667			
Total	41.8	19				

LEE	PCE	PD	R
5	5	3	4
3	5	5	5
5	4	4	5
	5	5	5
	3	4	5
	4		5

Anova: Single Factor

SUMMARY

Groups	Count	Sum	Average	Variance
LEE	3	13	4.33333333	1.33333333
PCE	6	26	4.33333333	0.66666667
PD	5	21	4.2	0.7
R	6	29	4.83333333	0.16666667

ANOVA

ırce of Variati	SS	df	MS	F	P-value	F crit
Between Gro	1.31666667	3	0.43888889	0.7289504	0.54958608	3.23887152
Within Grour	9.63333333	16	0.60208333			
Total	10.95	19				

LEE	PCE	PD	R
5	5	3	5
5	5	5	3
5	3	3	4
	3	5	4
	2	4	4
	4		5

Anova: Single Factor

SUMMARY

Groups	Count	Sum	Average	Variance
LEE	3	15	5	0
PCE	6	22	3.66666667	1.46666667
PD	5	20	4	1
R	6	25	4.16666667	0.56666667

ANOVA

ırce of Variati	SS	df	MS	F	P-value	F crit
Between Gro	3.63333333	3	1.21111111	1.36784314	0.28833571	3.23887152
Within Grour	14.1666667	16	0.88541667			
Total	17.8	19				

LEE	PCE	PD	R
5	5	2	5
5	5	5	4
5	4	5	5
	4	5	5
	4	3	4
	3		5

Anova: Single Factor

SUMMARY

Groups	Count	Sum	Average	Variance
LEE	3	15	5	0
PCE	6	25	4.16666667	0.56666667
PD	5	20	4	2
R	6	28	4.66666667	0.26666667

ANOVA

urce of Variati	SS	df	MS	F	P-value	F crit
Between Gro	2.63333333	3	0.87777778	1.1543379	0.35768581	3.23887152
Within Group	12.1666667	16	0.76041667			
Total	14.8	19				

LEE	*PCE*	*PD*	*R*
5	5	1	5
5	4	5	3
5	3	4	4
	2	5	4
	1	4	3
	2		5

Anova: Single Factor

SUMMARY

Groups	*Count*	*Sum*	*Average*	*Variance*
LEE	3	15	5	0
PCE	6	17	2.83333333	2.16666667
PD	5	19	3.8	2.7
R	6	24	4	0.8

ANOVA

urce of Variati	*SS*	*df*	*MS*	*F*	*P-value*	*F crit*
Between Gro	10.1166667	3	3.37222222	2.10489814	0.13983715	3.23887152
Within Group	25.6333333	16	1.60208333			
Total	35.75	19				

LEE	PCE	PD	R
5	5	4	4
3	5	4	5
5	4	3	4
	4	5	4
	3	3	5
	4		5

Anova: Single Factor

SUMMARY

Groups	Count	Sum	Average	Variance
LEE	3	13	4.33333333	1.33333333
PCE	6	25	4.16666667	0.56666667
PD	5	19	3.8	0.7
R	6	27	4.5	0.3

ANOVA

ırce of Variati	SS	df	MS	F	P-value	F crit
Between Gro	1.4	3	0.46666667	0.76190476	0.53178268	3.23887152
Within Group	9.8	16	0.6125			
Total	11.2	19				

LEE	*PCE*	*PD*	*R*
5	5	4	3
3	5	4	4
5	4	5	4
	4	4	4
	1	4	5
	4		5

Anova: Single Factor

SUMMARY

Groups	*Count*	*Sum*	*Average*	*Variance*
LEE	3	13	4.33333333	1.33333333
PCE	6	23	3.83333333	2.16666667
PD	5	21	4.2	0.2
R	6	25	4.16666667	0.56666667

ANOVA

urce of Variati	*SS*	*df*	*MS*	*F*	*P-value*	*F crit*
Between Gro	0.66666667	3	0.22222222	0.2075227	0.88969208	3.23887152
Within Grour	17.1333333	16	1.07083333			
Total	17.8	19				

LEE	*PCE*	*PD*	*R*
5	5	3	4
2	4	4	5
5	2	3	4
	5	4	4
	1	4	4
	4		5

Anova: Single Factor

SUMMARY

Groups	*Count*	*Sum*	*Average*	*Variance*
LEE	3	12	4	3
PCE	6	21	3.5	2.7
PD	5	18	3.6	0.3
R	6	26	4.33333333	0.26666667

ANOVA

ırce of Variati	*SS*	*df*	*MS*	*F*	*P-value*	*F crit*
Between Gro	2.51666667	3	0.83888889	0.60917801	0.61870408	3.23887152
Within Group	22.0333333	16	1.37708333			
Total	24.55	19				

LEE	PCE	PD	R
5	5	4	4
2	4	4	4
5	4	4	4
	3	5	4
	1	4	5
	4		5

Anova: Single Factor

SUMMARY

Groups	Count	Sum	Average	Variance
LEE	3	12	4	3
PCE	6	21	3.5	1.9
PD	5	21	4.2	0.2
R	6	26	4.33333333	0.26666667

ANOVA

urce of Variati	SS	df	MS	F	P-value	F crit
Between Gro	2.36666667	3	0.78888889	0.71581601	0.55682792	3.23887152
Within Group	17.6333333	16	1.10208333			
Total	20	19				

LEE	*PCE*	*PD*	*R*
5	5	4	5
3	4	4	5
5	4	5	4
	4	5	4
	4	4	5
	4		5

Anova: Single Factor

SUMMARY

Groups	*Count*	*Sum*	*Average*	*Variance*
LEE	3	13	4.33333333	1.33333333
PCE	6	25	4.16666667	0.16666667
PD	5	22	4.4	0.3
R	6	28	4.66666667	0.26666667

ANOVA

urce of Variati	*SS*	*df*	*MS*	*F*	*P-value*	*F crit*
Between Gro	0.76666667	3	0.25555556	0.67771639	0.57830662	3.23887152
Within Group	6.03333333	16	0.37708333			
Total	6.8	19				

LEE	*PCE*	*PD*	*R*
5	5	4	5
2	5	5	5
5	5	4	5
	4	5	4
	4	5	5
	5		5

Anova: Single Factor

SUMMARY

Groups	*Count*	*Sum*	*Average*	*Variance*
LEE	3	12	4	3
PCE	6	28	4.66666667	0.26666667
PD	5	23	4.6	0.3
R	6	29	4.83333333	0.16666667

ANOVA

urce of Variati	*SS*	*df*	*MS*	*F*	*P-value*	*F crit*
Between Gro	1.43333333	3	0.47777778	0.81613286	0.50361915	3.23887152
Within Grour	9.36666667	16	0.58541667			
Total	10.8	19				

LEE	*PCE*	*PD*	*R*
5	5	4	5
3	5	4	5
5	5	5	5
	3	5	4
	4	5	4
	4		5

Anova: Single Factor

SUMMARY

Groups	*Count*	*Sum*	*Average*	*Variance*
LEE	3	13	4.33333333	1.33333333
PCE	6	26	4.33333333	0.66666667
PD	5	23	4.6	0.3
R	6	28	4.66666667	0.26666667

ANOVA

ırce of Variati	*SS*	*df*	*MS*	*F*	*P-value*	*F crit*
Between Gro	0.46666667	3	0.15555556	0.29166667	0.83079496	3.23887152
Within Group	8.53333333	16	0.53333333			
Total	9	19				

LEE	*PCE*	*PD*	*R*
5	5	4	5
5	5	5	3
5	4	3	4
	4	5	4
	3	5	4
	5		5

Anova: Single Factor

SUMMARY

Groups	*Count*	*Sum*	*Average*	*Variance*
LEE	3	15	5	0
PCE	6	26	4.33333333	0.66666667
PD	5	22	4.4	0.8
R	6	25	4.16666667	0.56666667

ANOVA

urce of Variati	*SS*	*df*	*MS*	*F*	*P-value*	*F crit*
Between Gro	1.43333333	3	0.47777778	0.81613286	0.50361915	3.23887152
Within Grour	9.36666667	16	0.58541667			
Total	10.8	19				

LEE	*PCE*	*PD*	*R*
5	5	4	4
1	4	4	5
5	4	5	5
	4	4	4
	4	4	4
	4		5

Anova: Single Factor

SUMMARY

Groups	*Count*	*Sum*	*Average*	*Variance*
LEE	3	11	3.66666667	5.33333333
PCE	6	25	4.16666667	0.16666667
PD	5	21	4.2	0.2
R	6	27	4.5	0.3

ANOVA

ırce of Variati	*SS*	*df*	*MS*	*F*	*P-value*	*F crit*
Between Gro	1.4	3	0.46666667	0.5410628	0.66103823	3.23887152
Within Group	13.8	16	0.8625			
Total	15.2	19				

LEE	*PCE*	*PD*	*R*
5	5	5	5
5	5	5	5
5	2	5	4
	3	5	5
	4	5	4
	4		5

Anova: Single Factor

SUMMARY

Groups	*Count*	*Sum*	*Average*	*Variance*
LEE	3	15	5	0
PCE	6	23	3.83333333	1.36666667
PD	5	25	5	0
R	6	28	4.66666667	0.26666667

ANOVA

ırce of Variati	*SS*	*df*	*MS*	*F*	*P-value*	*F crit*
Between Gro	4.78333333	3	1.59444444	3.12380952	0.05523007	3.23887152
Within Grouյ	8.16666667	16	0.51041667			
Total	12.95	19				

LEE	*PCE*	*PD*	*R*
3	5	5	4
3	4	3	5
5	2	4	5
	4	4	4
	5	4	4
	3		5

Anova: Single Factor

SUMMARY

Groups	*Count*	*Sum*	*Average*	*Variance*
LEE	3	11	3.66666667	1.33333333
PCE	6	23	3.83333333	1.36666667
PD	5	20	4	0.5
R	6	27	4.5	0.3

ANOVA

urce of Variati	*SS*	*df*	*MS*	*F*	*P-value*	*F crit*
Between Gro	1.95	3	0.65	0.8	0.5118515	3.23887152
Within Group	13	16	0.8125			
Total	14.95	19				

LEE	PCE	PD	R
3	5	5	5
3	4	4	5
5	4	2	4
	1	4	4
	1	3	3
	4		5

Anova: Single Factor

SUMMARY

Groups	Count	Sum	Average	Variance
LEE	3	11	3.66666667	1.33333333
PCE	6	19	3.16666667	2.96666667
PD	5	18	3.6	1.3
R	6	26	4.33333333	0.66666667

ANOVA

ırce of Variati	SS	df	MS	F	P-value	F crit
Between Gro	4.16666667	3	1.38888889	0.85360649	0.48496971	3.23887152
Within Grour	26.0333333	16	1.62708333			
Total	30.2	19				

LEE	*PCE*	*PD*	*R*
5	5	5	4
3	5	5	5
5	5	5	5
	4	5	5
	4	4	2
	5		5

Anova: Single Factor

SUMMARY

Groups	*Count*	*Sum*	*Average*	*Variance*
LEE	3	13	4.33333333	1.33333333
PCE	6	28	4.66666667	0.26666667
PD	5	24	4.8	0.2
R	6	26	4.33333333	1.46666667

ANOVA

urce of Variati	*SS*	*df*	*MS*	*F*	*P-value*	*F crit*
Between Gro	0.81666667	3	0.27222222	0.35897436	0.78340236	3.23887152
Within Group	12.1333333	16	0.75833333			
Total	12.95	19				

LEE	*PCE*	*PD*	*R*
5	5	4	5
3	5	5	5
5	4	5	5
	4	5	4
	4	4	5
	4		5

Anova: Single Factor

SUMMARY

Groups	*Count*	*Sum*	*Average*	*Variance*
LEE	3	13	4.33333333	1.33333333
PCE	6	26	4.33333333	0.26666667
PD	5	23	4.6	0.3
R	6	29	4.83333333	0.16666667

ANOVA

urce of Variati	*SS*	*df*	*MS*	*F*	*P-value*	*F crit*
Between Gro	0.91666667	3	0.30555556	0.81031308	0.50657469	3.23887152
Within Group	6.03333333	16	0.37708333			
Total	6.95	19				

LEE	*PCE*	*PD*	*R*
5	5	4	5
1	5	5	5
5	5	5	4
	5	5	4
	5	5	5
	5		5

Anova: Single Factor

SUMMARY

Groups	*Count*	*Sum*	*Average*	*Variance*
LEE	3	11	3.66666667	5.33333333
PCE	6	30	5	0
PD	5	24	4.8	0.2
R	6	28	4.66666667	0.26666667

ANOVA

ırce of Variati	*SS*	*df*	*MS*	*F*	*P-value*	*F crit*
Between Gro	3.75	3	1.25	1.5625	0.23732527	3.23887152
Within Group	12.8	16	0.8			
Total	16.55	19				

LEE	*PCE*	*PD*	*R*
5	5	4	5
3	4	4	5
5	5	4	5
	4	4	5
	5	5	5
	5		5

Anova: Single Factor

SUMMARY

Groups	*Count*	*Sum*	*Average*	*Variance*
LEE	3	13	4.33333333	1.33333333
PCE	6	28	4.66666667	0.26666667
PD	5	21	4.2	0.2
R	6	30	5	0

ANOVA

urce of Variati	*SS*	*df*	*MS*	*F*	*P-value*	*F crit*
Between Gro	2	3	0.66666667	2.22222222	0.12509676	3.23887152
Within Group	4.8	16	0.3			
Total	6.8	19				

LEE	PCE	PD	R
5	5	5	5
4	5	4	5
5	5	5	5
	4	5	5
	5	5	5
	5		5

Anova: Single Factor

SUMMARY

Groups	Count	Sum	Average	Variance
LEE	3	14	4.66666667	0.33333333
PCE	6	29	4.83333333	0.16666667
PD	5	24	4.8	0.2
R	6	30	5	0

ANOVA

ırce of Variati	SS	df	MS	F	P-value	F crit
Between Gro	0.25	3	0.08333333	0.57971014	0.6367571	3.23887152
Within Group	2.3	16	0.14375			
Total	2.55	19				

LEE	*PCE*	*PD*	*R*
5	5	5	5
2	5	4	5
5	4	4	5
	4	5	5
	5	5	4
	5		5

Anova: Single Factor

SUMMARY

Groups	*Count*	*Sum*	*Average*	*Variance*
LEE	3	12	4	3
PCE	6	28	4.66666667	0.26666667
PD	5	23	4.6	0.3
R	6	29	4.83333333	0.16666667

ANOVA

urce of Variati	*SS*	*df*	*MS*	*F*	*P-value*	*F crit*
Between Gro	1.43333333	3	0.47777778	0.81613286	0.50361915	3.23887152
Within Grour	9.36666667	16	0.58541667			
Total	10.8	19				

LEE	*PCE*	*PD*	*R*
5	5	3	4
3	5	5	4
5	4	5	5
	4	5	4
	4	4	3
	4		5

Anova: Single Factor

SUMMARY

Groups	*Count*	*Sum*	*Average*	*Variance*
LEE	3	13	4.33333333	1.33333333
PCE	6	26	4.33333333	0.26666667
PD	5	22	4.4	0.8
R	6	25	4.16666667	0.56666667

ANOVA

ırce of Variati	*SS*	*df*	*MS*	*F*	*P-value*	*F crit*
Between Gro	0.16666667	3	0.05555556	0.08859358	0.96527532	3.23887152
Within Group	10.0333333	16	0.62708333			
Total	10.2	19				

LEE	*PCE*	*PD*	*R*
5	5	4	5
3	5	5	5
5	5	5	5
	5	5	5
	5	5	5
	4		5

Anova: Single Factor

SUMMARY

Groups	*Count*	*Sum*	*Average*	*Variance*
LEE	3	13	4.33333333	1.33333333
PCE	6	29	4.83333333	0.16666667
PD	5	24	4.8	0.2
R	6	30	5	0

ANOVA

urce of Variati	*SS*	*df*	*MS*	*F*	*P-value*	*F crit*
Between Gro	0.9	3	0.3	1.11627907	0.3717443	3.23887152
Within Group	4.3	16	0.26875			
Total	5.2	19				
